# Heterozygous deletion of 10q24.31-q24.33– a new syndrome associated with multiple congenital anomalies: case report and literature review

**DOI:** 10.1186/s42466-025-00378-z

**Published:** 2025-04-07

**Authors:** Anastasiia A. Buianova, Yulia S. Lashkova, Tatiana V. Kulichenko, Ivan S. Kuznetsov, Artem A. Ivanov, Olga P. Parshina, Oleg N. Suchalko, Svetlana S. Vakhlyarskaya, Dmitriy O. Korostin

**Affiliations:** 1 Center for Precision Genome Editing and Genetic Technologies for Biomedicine, Pirogov Russian National Research Medical University, 1, bld. 1, Ostrovityanova St., Moscow, 117513 Russia; 2 Russian Children’s Clinical Hospital, 117 Leninsky Prospect, Moscow, 119571 Russia

**Keywords:** Deletion, Copy number variation, Whole-exome sequencing, Developmental delay, Multiple congenital anomalies

## Abstract

**Background:**

Congenital anomalies and neurodevelopmental disorders are complex conditions often requiring comprehensive diagnostic approaches. Next-generation sequencing (NGS), particularly whole-exome sequencing (WES), has greatly improved the detection of pathogenic variants, including copy number variations (CNVs), which account for up to 35% of genetic causes in neurological patients. Combining CNV and single nucleotide variant (SNV) analysis through WES enhances diagnostic accuracy, especially in cases with unclassified congenital anomalies.

**Case presentation and literature review:**

This study reports a 14-year-old male patient with multiple congenital anomalies, including hypospadias, complete cleft palate, and recurrent pneumonia. His clinical presentation includes significant physical and intellectual developmental delays, autism-like symptoms, and spastic diplegia. Whole-exome sequencing (WES) was performed due to these complex symptoms, revealing a novel heterozygous deletion on chromosome 10q24.31-q24.33. Laboratory findings indicated agammaglobulinemia, leading to prophylactic antibiotic therapy and immunoglobulin replacement. Additional imaging studies showed cystic malformation of the middle lobe of the right lung, sliding hiatal hernia with prolapse of the gastric mucosa, and brain anomalies consistent with Joubert syndrome.

**Conclusions:**

This case underscores the importance of genetic analysis in understanding the etiology of congenital anomalies and neurodevelopmental disorders, providing critical insights into the molecular mechanisms driving complex phenotypes. The identified chromosomal deletion contributes to the existing literature on genomic imbalances associated with similar phenotypes.

## Background

The diagnostic potential for genetic disorders has been greatly increased by next-generation sequencing (NGS). A meta-analysis of 34,081 studies reveals a 36% diagnostic effectiveness for NGS in patients with neurodevelopmental disorders [1]. Chromosomal abnormalities are more prevalent than monogenic disorders in individuals with birth defects [2]. Whole-exome sequencing (WES) can discover 88% of pathogenic structural genomic variants (copy number variations, or CNVs) greater than three exons in length [1]. CNVs account for up to 35% of pathogenic variants in neurological patients, driven in part by genes such as *PMP22*, *SMN1* and *DMD* [3]. While conventional cytogenetic techniques typically fail to identify CNVs, these variations are more prevalent than single nucleotide variants (SNVs) [4], and WES can detect SNVs, even at low-level mosaicism [5]. Consequently, WES offers a deeper genetic analysis for patients with unclassified congenital anomalies, and combining CNV and SNV searches enhances its diagnostic power [6, 7]. Most CNV loci linked to neurodevelopmental disorders contain multiple genes highly expressed in the brain across developmental stages; the proteins they encode are involved in common pathogenic pathways [8]. Approximately 14.2% of multiple congenital anomalies and intellectual disabilities in patients are associated with CNVs over 400 kb [9]. Three patients had CNVs found in a recent WES investigation, which determined the etiology in 48.9% of instances [10].

This paper describes a 14-year-old male patient with congenital anomalies including hypospadias, complete cleft palate, and upper lip. The patient exhibited physical and intellectual developmental delays, autism-like symptoms, and cerebral palsy with spastic diplegia. The patient also had an immunodeficiency (agammaglobulinemia) and a history of recurrent pneumonias. This symptom complex led to WES analysis, revealing a previously unreported heterozygous deletion on the long arm of chromosome 10.

## Case presentation

**Fig. 1:**
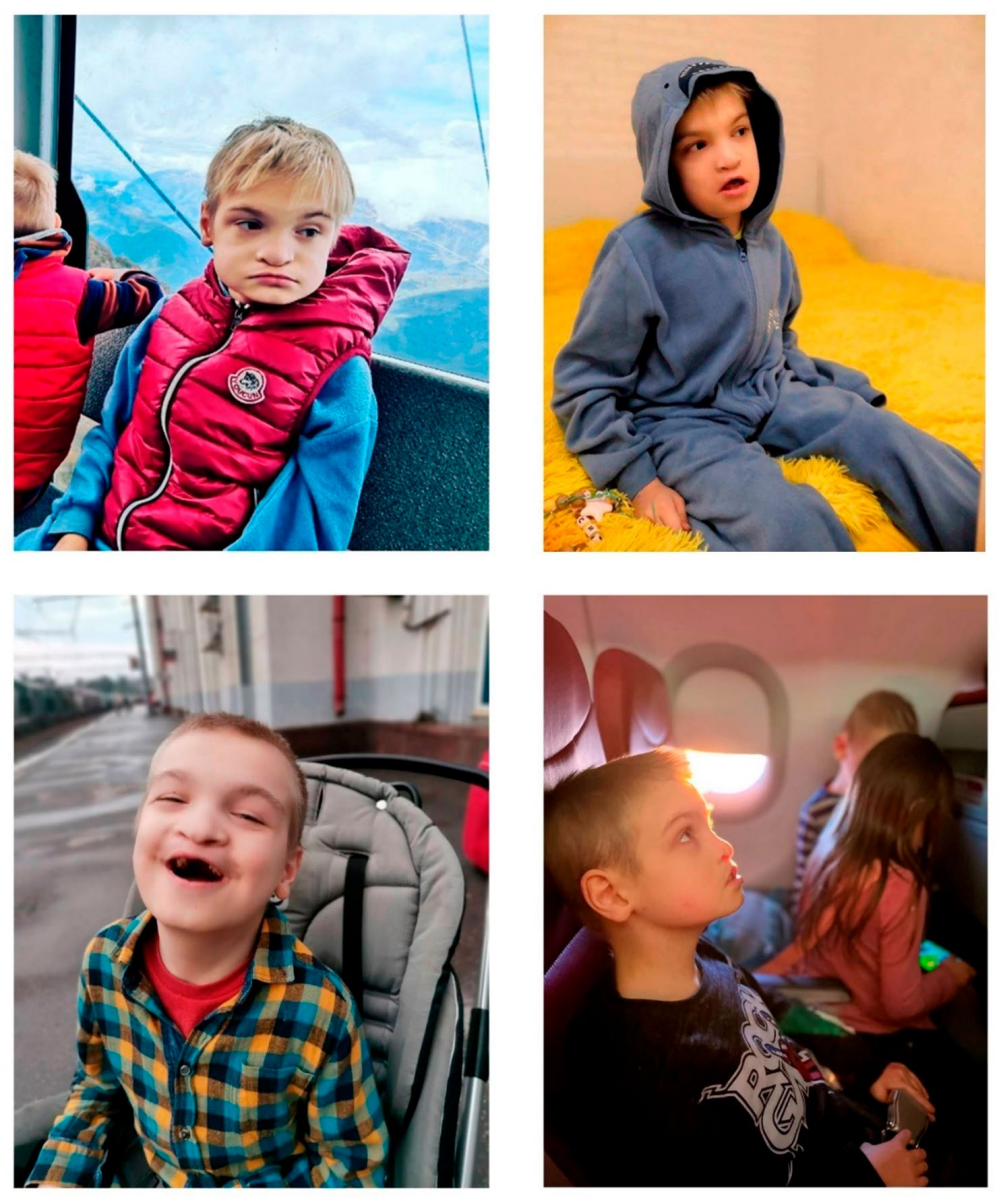
Patient’s appearance. Photo from the family archive

A 14-year-old male (Fig. 1) was admitted to the pediatric diagnostic department of the Russian Children’s Clinical Hospital, a branch of the Pirogov Russian National Research Medical University, for evaluation due to frequent pneumonia episodes. His mother has autoimmune thyroiditis. The child was born after the first pregnancy, complicated by two episodes of acute respiratory infection and hypertension in the mother. He was delivered at 40 weeks by vacuum extraction, with a birth weight of 3300 g and length of 52 cm. Apgar scores were 8/8.

From birth, the patient presented with multiple congenital anomalies, including hypospadias and complete clefts of the hard palate and upper lip. Psychomotor delay was noted from early infancy. Surgical correction of the cleft lip was performed at 1 year 4 months, and cleft palate repair at 5 years. Currently, he can walk with support, sits unaided, but does not speak or respond to simple commands. Diagnosed with cerebral palsy with spastic diplegia, his motor function level is classified as GMFCS III.

Over the past three years, he experienced approximately six pneumonia episodes.

In September 2023, at the age of 14, his parents brought him to the Russian Children’s Clinical Hospital for examination (hospitalized from 18.09.2023 to 08.10.2023). Upon admission, physical developmental delay was noted (height: 132 cm, <3rd percentile; weight: 24.5 kg, <3rd percentile, proportionate). Body temperature was normal. The patient did not speak, and did not follow simple commands, exhibiting severe intellectual disability. He could sit independently but required support to walk. Skin was pale without rashes; post-surgical scar on the lip was present. No edema; peripheral lymph nodes were unremarkable. Distal phalangeal deformities (clubbing) were noted. Respiration: 20/min, SpO₂: 98%. Nasal breathing was obstructed, with thick nasal discharge. Lung examination revealed vesicular breathing, diminished in the lower zones; no wheezing. Pulse: 90 bpm, regular rhythm, standing pulse rate: 90 bpm. Blood pressure (right arm): 100/70 mmHg. Heart sounds were rhythmic, clear, without murmurs. No appetite issues, occasional dysphagia with regurgitation. Abdomen: non-distended, soft, non-tender; liver and spleen not enlarged. Bladder and bowel function: incontinent. Stool: prone to constipation. Urination: free, painless, unchanged urine color, in diapers. The patient responded to auditory and visual stimuli.

Laboratory results showed agammaglobulinemia (IgG 0.31 g/L [normal 6.2–14.2], IgM 0.05 g/L [normal 0.5–1.7], IgA 0.01 g/L [normal 0.5–3]). Prophylactic therapy with trimethoprim-sulfamethoxazole and intravenous immunoglobulin (IVIG) replacement therapy were initiated.

Ophthalmologic examination revealed background retinopathy and concomitant divergent strabismus. Hypomagnesemia was also noted (serum magnesium level: 0.61 mmol/L, normal range: 0.74–1.2 mmol/L).

WES results were received on 19.10.2023. The patient was also evaluated by a geneticist at the Research Centre for Medical Genetics, where a blood sample was taken for karyotyping and chromosomal microarray analysis (CMA).

**Fig. 2:**
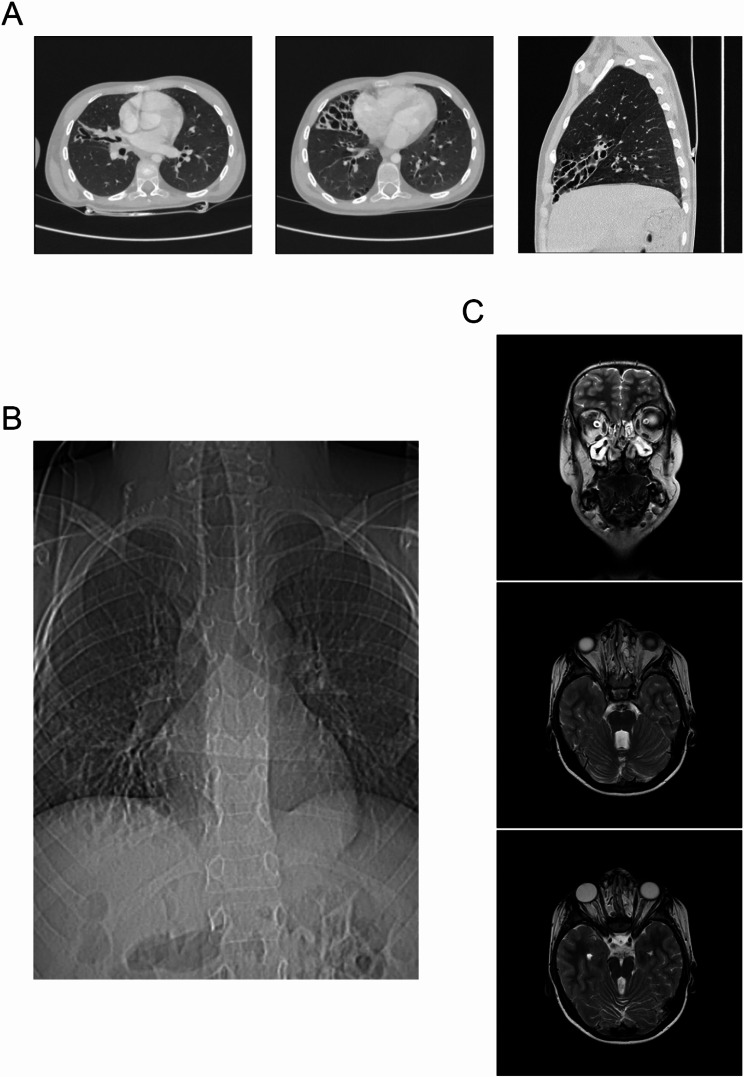
**(A)** Chest computed tomography scan showing cystic malformation of the middle lobe of the right lung, hiatal hernia with gastric prolapse, subpleural cysts, and bronchiectasis. **(B)** Esophageal and gastric fluoroscopy with barium contrast demonstrating a sliding hiatal hernia with prolapse of the gastric mucosa. **(C)** Brain magnetic resonance imaging indicating anomalies consistent with Joubert syndrome, cleft palate, and edema with polyposis of the paranasal sinuses

Results of the chest computed tomography in the child revealed changes characteristic of cystic malformation of the middle lobe of the right lung, a hiatal hernia with prolapse of a part of the stomach, multiple subpleural cysts, and bronchiectasis (Fig. 2A).

Results of the fibroesophagogastroduodenoscopy showed gastroesophageal reflux in the patient. High gastroesophageal reflux (up to the upper third of the esophagus), as well as a sliding hiatal hernia with prolapse of the gastric mucosa into the esophagus, were confirmed by radiography of the esophagus and stomach with barium contrast (Fig. 2B). Based on the examination, a gastrostomy and Nissen fundoplication have been planned.

The small intestine biopsy characterizes signs of mildly active duodenitis, including deformed villi. The gastric biopsy reveals reactive gastropathy with foveolar hyperplasia of the glands. The lamina propria contains moderate lymphoplasmacytic infiltration with a mixture of neutrophils and eosinophils in both the stomach and small intestine. H. Pylori is not detected in Giemsa staining. The esophagus exhibits changes characteristic of esophagitis, with lymphocytic infiltration, mild basal layer hyperplasia, spongiosis, and increased height of connective tissue papillae.

Magnetic resonance imaging (MRI) of the brain showed brain anomalies consistent with Joubert syndrome. A cleft palate, an open nasopharyngeal fistula, and edema with polyposis of the nasal sinuses were identified (Fig. 2C). The child has been consulted by a maxillofacial surgeon, and surgical correction of the cleft palate has been planned.

Management Recommendations:

- Regular IVIG replacement therapy to prevent infectious complications.
- Prophylactic antibiotics (trimethoprim-sulfamethoxazole, Biseptol^®^, 480 mg twice a day) for immunodeficiency-related infections.
- Surgical procedures to correct anatomical defects and enhance breathing and eating.
- Magnesium supplementation due to *CNNM2*-related hypomagnesemia.
- Long-term neurodevelopmental and ophthalmologic follow-up given the involvement of *ARL3* (retinitis pigmentosa) and *SUFU* (medulloblastoma risk).

## Genetic testing

Genomic DNA was extracted from venous blood using the QIAamp DNA Blood Mini Kit (Qiagen, Hilden, Germany). DNA libraries were prepared from 500 ng of genomic DNA using the MGI Easy Universal DNA Library Prep Set (MGI Tech, Shenzhen, China) per manufacturer’s protocol. DNA fragmentation was performed using ultrasonication on a Covaris S-220 (Covaris, Inc., Woburn, MA, USA), resulting in an average fragment length of 250 bp. DNA library enrichment was done by pre-pooling using SureSelect Human All Exon v8 probes (Agilent Technologies, Santa Clara, CA, USA), covering the full human exome [11]. DNA and library concentrations were measured with a Qubit Flex (Life Technologies, Carlsbad, CA, USA) using the dsDNA HS Assay Kit. Library quality was assessed using the Bioanalyzer 2100 with High Sensitivity DNA Kit (Agilent Technologies, Santa Clara, CA, USA). The libraries were circularized and sequenced in paired-end mode on a DNBSEQ G-400 platform using the DNBSEQ-G400RS High-throughput Sequencing Set PE100 (MGI Tech, Shenzhen, China), with an average coverage of 100×. FastQ files were generated using MGI Tech’s basecallLite software (ver. 1.0.7.84) from the manufacturer (MGI Tech, Shenzhen, China).

Bioinformatics quality control and sequencing data correction were performed using FastQC v0.11.9 [12] and bbduk v38.96 [13]. Alignment to the human reference genome GRCh37 was carried out with bwa-mem2 v2.2.1 [14]. Aligned files were converted to BAM format, sorted, and indexed with SAMtools v1.9 [15]. Duplicates were marked and quality metrics were collected with Picard v2.22.4 [16]. Variant calling was performed with bcftools v1.9 [17] and deepvariant v1.5.0 [18]. CNV analysis was conducted with CNVkit [19], ClinViewer [20], and annotated with AnnotSV [21]. Clinical significance assessment followed ACMG guidelines [22, 23]. The identified variant was validated by CMA using Affimetrix CytoScan HD arrays on a venous blood sample.

A heterozygous deletion spanning 2.1 Mb (10q24.31-q24.33) on chromosome 10 was detected, involving 38 protein-coding genes, 12 of which (*ARL3*, *CNNM2*, *CYP17A1*, *FGF8*, *GBF1*, *HPS6*, *LBX1*, *NFKB2*, *NT5C2*, *PITX3*, *SUFU*, *TRIM8*) are disease-associated. Germline mutations in *ARL3* are associated with Retinitis pigmentosa 83 (OMIM: 618173); *CNNM2* is linked to Hypomagnesemia 6, renal (OMIM: 613882) and Hypomagnesemia, seizures, and impaired intellectual development 1 (OMIM: 616418). *FGF8* mutations are linked to Hypogonadotropic hypogonadism 6 with or without anosmia (OMIM: 612702); *GBF1* mutations associate with Charcot-Marie-Tooth disease, axonal, type 2GG (OMIM: 606483); *NFKB2* mutations with Immunodeficiency, common variable, 10 (OMIM: 615577); *PITX3* mutations with Anterior segment dysgenesis 1, multiple subtypes (OMIM: 107250) and Cataract 11, multiple types (OMIM: 610623). *SUFU* mutations are linked to Medulloblastoma (OMIM: 155255), familial meningiomas (OMIM: 607174), and Basal cell nevus syndrome 2 (OMIM: 620343). *TRIM8* mutations are associated with Focal segmental glomerulosclerosis and neurodevelopmental syndrome (OMIM: 619428). Autosomal recessive syndromes and genes associated solely with this inheritance pattern are excluded, as no second clinically significant allele was detected.

**Fig. 3:**
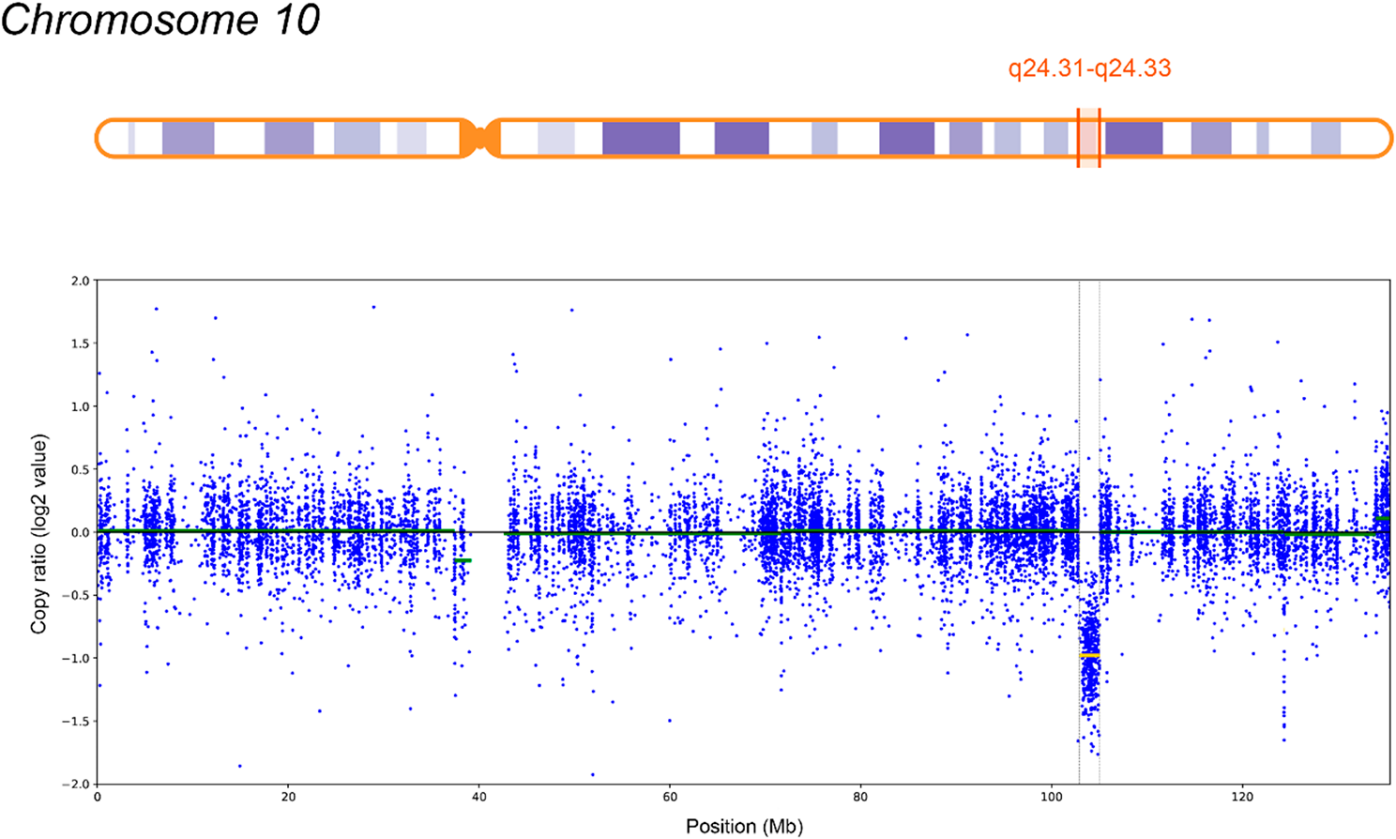
Region of heterozygous copy loss arr[GRCh37] 10q24.31-q24.33(102837530_105033440)x1 (∼ 2.2 Mb) of patient N., displayed in the IGV genome browser (data from whole exome sequencing)

The identified CNV is not associated with known microdeletion syndromes and has not been reported in a healthy population. According to AnnotSV, GC content is 63.0% and 67.0% within ± 100 bp of the CNV boundaries, suggesting potential non-allelic homologous recombination. The ExAC Z-score for deletions is lower than for duplications (1.093 and 1.384, respectively), indicating that the region is less tolerant to copy number increase. Based on ACMG criteria 2 A (+ 1), 3 C (+ 0.9), and 4 L (+ 0.1), the deletion was classified as pathogenic (total score: 2). Validation by CMA (Fig. 3): Molecular Karyotype (ISCN 2020): arr[GRCh37] 10q24.31-q24.33(102837530_105033440)x1 (∼ 2.2 Mb), closely matching WES findings. The total length of regions of loss of heterozygosity, measuring 3 million bp or more, corresponds to the population rate (0.55%).

The final list of genes included in the deletion region: *ACTR1A*, *ARL3*, *ARMH3*, *AS3MT*, *BORCS7*, *BORCS7-ASMT*, *BTRC*, *C10orf95*, *CNNM2*, *CUEDC2*, *CYP17A1*, *DPCD*, *ELOVL3*, *FBXL15*, *FBXW4*, *FGF8*, *GBF1*, *HPS6*, *KCNIP2*, *KCNIP2-AS1*, *LBX1*, *LBX1-AS1*, *LDB1*, *LINC01514*, *LINC02681*, *LOC107984265*, *MFSD13A*, *MIR146B*, *MIR3158-1*, *MIR3158-2*, *NFKB2*, *NOLC1*, *NPM3*, *NT5C2*, *OGA*, *PITX3*, *POLL*, *PPRC1*, *PSD*, *RPARP-AS1*, *RPEL1*, *SFXN2*, *SUFU*, *TLX1*, *TLX1NB*, *TRIM8*, *WBP1L* (total of 47); of which 37 encode proteins. Genes associated with diseases were identical to those found on WES. The haploinsufficiency genes are *ACTR1A*, *CNNM2*, *LDB1*, *NFKB2*, *PPRC1*, *PSD*, *SUFU*, *TRIM8*, *FGF8*, *WBP1L*, *BTRC*, *LBX1*, *NT5C2*, *PITX3* (according to LOEUF, pLI, pHI, %HI), and the triplosensitive genes are *LDB1* and *TRIM8* (according to pTS). In the UK Biobank v2, 6 deletions and 36 duplications were detected in this region. In gnomAD SVs v4.1.0, the 5 largest deletions with a frequency of 0.000008 (per allele) ranged in size from 340 to 2041.53 kb, 4 of which had a LOF effect, and 1 was located in the intron of *CNNM2*. Despite the detection of 569 deletions and only 266 duplications, the largest 10 deletions were in the range of 0.158–0.745 Mb. This region also contained 239 insertions (ranging from 50 bp to 6017 bp) and 5 inversions.

The identified CNV has been registered in ClinVar: https://www.ncbi.nlm.nih.gov/clinvar/variation/3342255/ (available online).

## Discussion

The heterozygous 10q24.31-q24.33 deletion identified in our patient encompasses 38 protein-coding genes, several of which are associated with neurodevelopmental disorders, primary immunodeficiency, and metabolic abnormalities. The clinical presentation of this particular deletion is similar to those of Joubert syndrome, NFKB2-associated primary immunodeficiency (CVID10), and *CNNM2*-related hypomagnesemia syndromes, although there isn’t a single recognized diagnosis that matches it. Early immunological evaluation and timely beginning of IVIG replacement therapy were essential in averting additional problems due to the severe agammaglobulinemia and recurrent pneumonia episodes. A multidisciplinary approach combining neurologists, geneticists, gastroenterologists, immunologists, and pulmonologists is required because of the patient’s severe intellectual disability, motor impairment, and congenital defects, which include cleft palate and hypospadias.

Despite the uneven coverage of target regions, unlike the uniform coverage achieved with whole genome sequencing (WGS), it is recommended to perform CNV analysis on exome data before resorting to WGS, which is approximately four times more expensive [24, 25]. For example, among 3040 patients who underwent WES, CNVs were found in 2.4% of cases, varying from a few exons of a single gene (1.4 Mb) to several genes (21.6 Mb) [26]. It is easier to identify large deletions on WES: heterozygous states exhibit runs of homozygosity, while homozygous states show no reads in the area. Duplications, especially when the syndrome has not been previously described and is not obvious from the clinical picture, are more challenging, as are instances of random read removal preceding the creation of the.bam file, when the variant allele frequency may change and abnormal genotypes may not be suspected. This limitation still exists; in one of the latest studies using WES data, 171 CNVs were identified, of which 140 were deletions and 15 duplications, while others represented more complex structural variants [27]. Analysis of WES data using ExomeDepth established that the sensitivity of the method is 89% for detecting large deletions and 65% for duplications, with a diagnostic value of 1.6%, which was comparable to CMA [28]. The CNVkit used in our practice differs from ExomeDepth and its counterparts by integrating sequence data from both target and non-target regions, thus providing a more comprehensive and accurate identification of CNVs.

The malformation ‘split hand/foot malformation’ (SHFM) is defined by median clefts of the hands and feet, aplasia and/or hypoplasia of the phalanges, metacarpal and metatarsal bones, as well as syndactyly. This rare condition occurs in 1 in 8500–25,000 newborns, yet accounts for 15% of all limb developmental disorders [29]. The most well-known condition associated with the locus 10q24 is SHFM type 3 (SHFM3) [30, 31], which is characterized by an increase in tandem genomic repeats of size 325–570 kb involving at least the gene *DACTYLIN* (*FBXW4*) [30]. The etiology of SHFM3 is associated with disruptions in the apical ectodermal ridge of the limbs. The *SHFM3* locus on 10q24 is conserved among vertebrates from zebrafish to humans, especially the region from *TLX1* to *FGF8*, and contains numerous regulatory elements active in mouse embryos. In the human genome, the highest density of conserved non-coding elements is found in *BTRC*. It is claimed that duplication of the first exon of the *BTRC* gene is responsible for the formation of the SHFM3 phenotype, possibly through cis- or trans-acting effects on genes or regulatory sequences involved in limb development [32]. There are described cases of SHFM3 that are not caused by duplications; for example, an 80.2 kb microdeletion in the 10q24.32 region was found in a girl with absence of the right radius and thumb, shortening of the right ulna, hypoplasia of the left thenar, and a small apical defect in the interventricular septum. This deletion included part of the *DPCD* gene (exons 2–6), all of *MIR3158-1*, *MIR3158-2*, and part of the *FBXW4* gene (exons 4–9). The CNV was inherited from an asymptomatic mother [33]. An analysis of the DECIPHER database conducted by Li CF et al. identified two male patients with deletions in the 10q24.31–q24.32 region. The CNV of the first patient measured 546.42 kb and included 13 genes from *LINC01514* to part of *FBXW4*, with the patient having a cleft palate and lip, intellectual disability, microphthalmia, kidney pathology, vesicoureteral reflux, pulmonary artery stenosis, sensorineural hearing loss, short stature, and abdominal obesity. The deletion size of the second patient was 389.02 kb, including genes from *BTRC* to part of *FBXW4*, and the patient had aortic dilation, Pierre Robin sequence, median cleft palate, kidney hypoplasia, and a secondary atrial septal defect [32]. One patient with bilateral radial dysplasia had a substitution g.103,380,009 A > G [hg19], or *FBXW4*(NM_022039.4):c.1301 + 4493T > C (6 intron), which had unclear clinical significance [33].

The literature presents cases of deletions of the long arm of chromosome 10 with close coordinates. A patient with a deletion of 3.79 Mb in the 10q24.2-q24.32 region was born with a left-sided cleft lip, nystagmus, and clubfoot. Subsequently, breathing problems, a congenital type I posterior urethral valve, vesicoureteral reflux of grades II-III, as well as polycystic and hypoplastic kidneys were diagnosed, eventually requiring peritoneal dialysis at the age of 4. The patient also experienced delayed descent of the testicles, significant vision loss, and hypoplasia of the corpus callosum [34]. Another patient with a 5.54 Mb deletion in 10q24.31–q25.1 exhibited bilateral cleft lip and palate, as well as Dandy-Walker malformation with bilateral ventriculomegaly. Postnatal MRI of the brain showed absence of the lower part of the cerebellar vermis, enlarged fourth ventricle opening into the midline retrocerebellar cystic space, increased posterior cranial fossa, and anomalies corresponding to lobar holoprosencephaly. The patient experienced developmental delays, spastic quadriplegia, and malnutrition, requiring the placement of a gastrojejunostomy tube and ventriculoperitoneal shunting. Despite rehabilitative therapy and surgical interventions, by 22 months, the patient could not sit up or turn over independently and exhibited delays in speech and motor skills [35].

In the DECIPHER clinical data database, smaller deletions fully encompassed within the deletion region are described. Among them, excluding other genetic variants, there were 3 girls (the age of only one is known: 6 years) and 6 boys (from infancy to 19 years), while the phenotype was unspecified in 1 girl and 1 boy. Nervous system disorders were found in five patients and manifested as global developmental delays (2/7), intellectual disability (2/7), absence/delay in speech (2/7), ataxia together with generalized hypotonia (2/7), and seizures with frontoparietal polymicrogyria (1/7). Two patients exhibited short stature, and one of them also had abdominal obesity with microphthalmia, cleft lip and palate, short phalanges, pulmonary artery stenosis, kidney anomalies, vesicoureteral reflux, and sensorineural hearing loss. Strabismus along with a high-arched palate was found in one patient, and MRI of the brain showed anomalies in cortical gyration, pyramidal signs, hypoplasia of the cerebellar vermis, brain atrophy, lobar holoprosencephaly, and partial agenesis of the corpus callosum. One patient had only joint contracture in the upper limbs.

FGF8 (fibroblast growth factor 8) regulates processes of cell proliferation, differentiation, and migration. It is necessary for the normal formation of the brain, eyes, ears, and limbs, as well as for the development of the neural system associated with the release of gonadotropin-releasing hormone [36]. According to epidemiological data from 23 regions of the Russian Federation from 2011 to 2018, cleft lip/palate occurs in 8.58 per 10,000 newborns (compared to 8.42 according to EUROCAT) [37], making it the most common craniofacial defect overall [38], and the risk of stillbirth for patients with this defect is relatively low: 10 in 1000 pregnancies [39]. However, establishing the causes of non-syndromic orofacial clefts is more challenging; WES managed this in 10% of a cohort of 84 patients [40]. We believe that the cleft lip and palate in our patient, along with the disorder of sexual development, are caused by the haploinsufficiency of *FGF8* [41]. It is hypothesized that the *ACTR1A* gene may be associated with intellectual disability since the dynactin it encodes functions within the presynaptic membrane to promote synaptic stability [42].

Loss-of-function mutations in the *NFKB2* gene, especially in its C-terminal domain, play a key role in the development of primary immunodeficiencies. The most frequently described amino acid residue variations occur at Arg853, leading to the formation of premature termination codons, while the transcripts are predicted to escape nonsense-mediated decay mechanisms. The truncated proteins lack phosphorylation sites necessary for interaction with NIK, resulting in the protein p100 not being converted to p52. Consequently, this disrupts the non-canonical NF-κB pathway required for normal B-cell function and antibody production, leading to a clinical picture of common variable immunodeficiency in early childhood [43]. One of the main complaints in the patient was recurrent pneumonias, likely of aspiration nature, although immunoglobulin levels A, M, and G were significantly reduced. We suspect that the deletion of the *NFKB2* gene is the cause of the patient’s immunodeficiency.

Uncommon structural variations may result in intricate multisystem illnesses, even without a clearly delineated diagnosis. In instances of multi-gene deletions, meticulous phenotypic analysis is crucial, considering both dominant and recessive disease causes. Extensive genetic testing, encompassing WES and CMA, ought to be contemplated promptly in the diagnostic procedure for sufferers exhibiting syndromic characteristics of indeterminate origin. Several clinical red flags can aid in early recognition of underlying genetic disorders. Frequent pneumonia episodes from childhood may indicate primary immunodeficiency, such as agammaglobulinemia. The presence of congenital anomalies, including cleft palate, hypospadias, and microcephaly, suggests an underlying genetic syndrome. A thorough neurogenetic and metabolic assessment is required for severe intellectual disability and psychomotor delay. Clubbing of the fingers, together with recurrent respiratory infections, increases concern for bronchiectasis or chronic lung disease.

Functional immunological investigations, such as T-cell proliferation assays and lymphocyte subpopulation analysis, may offer additional understanding of immune dysfunction to enhance the diagnostic work-up. Furthermore, RNA sequencing could aid in describing how the identified loss affects gene expression. To monitor the course of the condition and improve supportive therapy, longitudinal neurodevelopmental evaluations might be helpful. Given the involvement of *FGF8*, which is associated with hypogonadotropic hypogonadism, an exhaustive endocrine evaluation is also recommended.

## Conclusions

Establishing an accurate genetic diagnosis in patients with multiple congenital anomalies and intellectual disabilities often poses a complex clinical challenge. Thus, our case demonstrates the importance of employing a comprehensive genetic approach (WES and CMA). The identified heterozygous deletion on chromosome 10q24.31-q24.33 is newly described and clinically significant, explaining the phenotype of this patient.

## Data Availability

The sequence data are generated from patient sample and therefore are available under restricted access.

## Abbreviations

ACMG: American College of Medical Genetics
bp: Base pair
CMA: Chromosomal Microarray Analysis
CNV: Copy Number Variation
GC: Guanine-Cytosine
GMFCS: Gross Motor Function Classification System
gnomAD: The Genome Aggregation Database
GRCh37: Genome Reference Consortium Human Build 37 (same as hg19)
HI: Haploinsufficiency
Ig: Immunglobulin
IGV: Integrative Genomics Viewer
ISCN: The International System for Human Cytogenomic Nomenclature
IVIG: Intravenous Immunoglobulin
LOEUF: Loss-of-function observed/expected upper bound fraction
LOF: Loss-of-function
MRI: Magnetic Resonance Imaging
NGS: Next-Generation Sequencing
OMIM: Online Mendelian Inheritance in Man
pHI: Predicts the Haploinsufficiency probability
pLI: Score indicating gene intolerance to a loss of function variation
pTS: Predicts the Triplosensitivity Scores
SHFM: Split Hand/Foot Malformation
SNV: Single Nucleotide Variant
WES: Whole-Exome Sequencing
